# Melatonin Reduces Mito‐Inflammation in Ischaemic Hippocampal HT22 Cells and Modulates the cGAS–STING Cytosolic DNA Sensing Pathway and FGF21 Release

**DOI:** 10.1111/jcmm.70285

**Published:** 2024-12-20

**Authors:** Silvia Carloni, Maria Gemma Nasoni, Anna Casabianca, Chiara Orlandi, Loredana Capobianco, Giorgia Natalia Iaconisi, Liana Cerioni, Sabrina Burattini, Serena Benedetti, Russel J. Reiter, Walter Balduini, Francesca Luchetti

**Affiliations:** ^1^ Department of Biomolecular Sciences University of Urbino Carlo Bo Urbino Italy; ^2^ Laboratorio Covid University of Urbino Carlo Bo Fano Italy; ^3^ Department of Biological Science and Technology University of Salento Lecce Italy; ^4^ Department of Cell Systems and Anatomy, Long School of Medicine UT Health San Antonio Texas USA

**Keywords:** FGF‐21, HT22, melatonin, mito‐inflammation, mtDNA, oxygen–glucose deprivation

## Abstract

Mitochondrial dysfunction is a key event in many pathological conditions, including neurodegenerative processes. When mitochondria are damaged, they release damage‐associated molecular patterns (DAMPs) that activate mito‐inflammation. The present study assessed mito‐inflammation after in vitro oxygen–glucose deprivation as a representation of ischaemia, followed by reoxygenation (OGD/R) of HT22 cells and modulation of the inflammatory response by melatonin. We observed that melatonin prevented mitochondrial structural damage and dysfunction caused by OGD/R. Melatonin reduced oxidative damage and preserved the enzymatic activity for complexes I, III and IV, encoded by mitochondrial DNA, which were reduced by OGD/R. No effect was observed on complex II activity encoded by nuclear DNA. The release of mtDNA into the cytosol was also prevented with a consequent reduction of the cGAS–STING pathway and IFNβ and IL‐6 production. Interestingly, melatonin also increased the early release of the fibroblast growth factor‐21 (FGF‐21), a mitokine secreted in response to mitochondrial stress. These data indicate that melatonin reduces mito‐inflammation and modulates FGF‐21 release, further highlighting the key role of this molecule in preserving mitochondrial integrity in OGD/R deprivation‐type ischaemic brain injury.

## 1 Introduction

Sterile inflammation is the immune response to endogenous molecules, defined as damage‐associated molecular patterns (DAMPs), released by cells in stress‐damaging conditions and the absence of pathogens [1]. Once released, DAMPs are recognised by pattern recognition receptors that activate complex downstream signalling cascades, releasing cytokines and initiating the inflammatory process [2]. Mitochondria, the energy‐producing organelles of cells, when damaged, subjected to stress or during physiological aging, release DAMPs, resulting in the activation of an inflammatory response. This process, recently defined as mito‐inflammation, identifies mitochondria as central regulators of the sterile inflammatory process [3]. Mito‐inflammation occurs in various neurodegenerative diseases, representing an early response leading to the inflammatory reaction [4]. Mitochondrial DNA (mtDNA) released into the cytoplasm is essential to this early response. In healthy mitochondria, mtDNA is distributed within the mitochondrial matrix and is crucial for their functions because it encodes subunits of the oxidative phosphorylation (OXPHOS) complexes. Since it lacks protective histones and is localised near the OXPHOS complexes, the major site of reactive oxygen species (ROS) formation, mtDNA is particularly susceptible to damage [5]. Accumulation of damaged mtDNA in mitochondria plays a key role in the progression of many neurodegenerative disorders [6, 7].

In acute brain injury, as during cerebral ischaemia/reperfusion injury, the high production of ROS can easily damage mtDNA, causing its release into the cytosol, as whole or in fragments, via the mitochondrial permeability transition pores (mPTP) [8]. Once released, mtDNA acts as a DAMP and activates the cGAS (cyclic GMP–AMP synthase)–STING signalling pathway [9]. STING activates TANK‐binding kinase 1 (TBK1), an important node protein for multiple signalling pathways implicated in autophagy, insulin signalling or cellular proliferation; TBK1 plays a key role in the immune innate response by coordinating the activation of interferon regulatory factor 3 (IRF) and NF‐κB and increasing the expression of type I interferon (IFN) and TNFα [10].

In response to stress, mitochondria also release mitokines. Fibroblast growth factor 21 (FGF‐21) is a mtDNA‐derived mitokine encoded by the MT‐RNR2 gene [11]. FGF21 is a stress‐related mitokine expressed in different tissues, including skeletal muscle, heart and brain, and released as a result of mitochondrial dysfunction [12]. FGF21 is also rapidly induced by fasting and mediates critical aspects of the adaptive starvation response [12]. FGF21 is considered a biomarker for mitochondrial diseases in skeletal muscle [13], but its expression and function in brain cells are still unknown. Increased serum FGF21 levels have been correlated with impaired OXPHOS and reduced ATP production and described as the response of cells to metabolic stress [14]. FGF21 also attenuates neurodegeneration by reducing neuroinflammation and oxidative stress by regulating NF‐kB and AMPK [15].

Melatonin (N‐acetyl‐5‐methoxytryptamine), an evolutionarily conserved ubiquitous distributed and acting molecule, has been widely recognised as a broad‐spectrum antioxidant and a potent free radical scavenger, which is synthesised in, taken up by, and concentrated in mitochondria [16, 17]. We previously found that melatonin effectively reduced mitochondrial ROS production, increased PGC1α and SIRT3 expressions, and reshaped the mitochondrial network, fostering mitochondrial transfer between ischaemic‐challenged HT22 cells through tunnelling nanotube connections [18]. By increasing the levels of SIRT3, melatonin also promotes the deacetylation of SOD2, thereby augmenting its function and aiding in the preservation of mitochondrial redox balance [19, 20].

The present study assessed mito‐inflammation in HT22 cells subjected to in vitro ischaemia followed by reoxygenation using the OGD/R model and how melatonin modulates this response. We show here that melatonin effectively reduces oxidative damage and mtDNA cytosolic release in HT22 cells following OGD/R, leading to decreased activation of the cGAS–STING pathway and reduced IFNβ gene and IL‐6 production. We also report that melatonin promotes FGF‐21 release in culture medium, suggesting the involvement of FGF‐21 in its neuroprotective effects.

## 2 Materials and Methods

### 2.1 Cell Culture

Murine hippocampal HT22 cells were cultured in DMEM‐HAM'S F12, supplemented with 10% foetal bovin serum, L‐glutamine (2 mM) and 1% antibiotics (penicillin, streptomycin). The cells were incubated in a humidified 5% CO_2_ atmosphere at 37°C. At 80% confluence, cells were detached with trypsin–EDTA, washed and sub‐cultivated in new 25cm^2^ flasks for 1–2 days before the experiments.

### 2.2 Simulation of In Vitro Ischaemia With OGD/R and Cell Treatments

Hypoxia–ischaemia was simulated by inducing transient oxygen–glucose deprivation followed by reoxygenation (OGD/R) as previously described [18]. Briefly, the cells were seeded at a density of 1 × 10^5^ cells/mL and incubated for 24 h to allow them to adhere. HT22 cells were maintained in the glucose‐free culture medium and then transferred into a temperature‐controlled (37°C) anaerobic chamber (Billups‐Rothenberg Modular Incubator chamber) containing a gas mixture composed of 5% CO_2_ and 95% N_2_. They were kept in the chamber for 8 h. Subsequently, the medium was replaced with normal DMEM containing glucose and the HT22 were returned to a normoxic condition for reoxygenation under 5% CO_2_/95% air. Controls were incubated with normal DMEM containing glucose in a humidified incubator with 5% CO_2_/95% air at 37°C for the same times as the OGD/R cultures.

### 2.3 Drug Treatment

Melatonin (Sigma‐Aldrich, Milan, Italy, M5250), dissolved in dimethyl sulfoxide (DMSO; Sigma‐Aldrich, D5879) and diluted in normal saline solution to a final concentration of 5% DMSO (vehicle), was added at a dose of 50 μM to the medium immediately after the OGD procedure and maintained at 37°C for 15, 30 min or 1 or 2 h (OGD/R). This dose of the drug was used based on previous experiments that showed the protective effects of melatonin in HT22 cells in OGD/R condition [18].

### 2.4 Oxyblot Assay

The protein carbonyl content is widely used as a marker for oxidative stress. Carbonylated proteins were detected by using the OxyBlot Protein Oxidation Detection Kit (Merck Millipore, Billerica, MA, USA) according to the manufacturer's instructions. Proteins obtained from each sample in equal amounts were split into two aliquots, each one underwent denaturation using a 6% SDS (w/v) solution. Subsequently, one aliquot was derivatised with 2,4‐dinitrophenylhydrazine (DNPH) solution, while the other aliquot was treated with a derivatisation‐control solution, serving as a non‐derivatised control. The derivatised and non‐derivatised proteins were then separated using 12% SDS‐PAGE and transferred onto a nitrocellulose membrane. After blocking non‐specific binding, the membrane was incubated with an anti‐dinitrophenyl primary antibody (1:150, polyclonal; Merck Millipore, Billerica, MA, USA, 90451) dilution in Tris‐buffered saline (TBS), 0.1% Tween‐20, 5% milk, at room temperature for 1 h. Following washing, the membrane underwent additional incubation with goat anti‐rabbit secondary antibody (Merck Millipore, Billerica, MA, USA, 90452) diluted 1:300 in TBS, 0.1% Tween‐20, 5% milk, at room temperature for 1 h. Immunoreactive protein bands were visualised using ECL chemiluminescence substrate and the ChemiDoc Imaging System (Bio‐Rad, Hercules, CA, US). The intensities of oxidised protein bands in each lane were quantified using Image Lab software (version 6.1 Bio‐Rad, Hercules, CA, US).

### 2.5 Spectrophotometric Measurement of Mitochondrial Respiratory Chain Complexes

Enzyme activity for mitochondrial respiratory chain complexes was quantified according to a previously published protocol [21]. Briefly, complex I activity was assessed in an assay mixture composed of 25 mM of potassium phosphate (pH 7.8), 3.5 g/L of bovine serum albumin (BSA), 80 μM of 2,6‐dichloroindophenol, 70 μM of decyl‐ubiquinone, 2 mM of EDTA, 10 μM of antimycin A and 0.2 mM of nicotinamide adenine dinucleotide (NADH). The inhibition of complex I was achieved by using 10 μM rotenone. The calculation of complex I activity involved measuring the linear decrease in 2,6‐dichloroindophenol absorbance at 600 nm over a 5‐min period.

For complex II, the assay mixture consisted of 80 mM of potassium phosphate (pH 7.8), 1 g/L of BSA, 60 μM of 2,6‐dichloroindophenol, 50 μM of decyl‐ubiquinone, 2 mM of EDTA, 10 μM of antimycin A and 20 mM of succinate. Inhibition of complex II was achieved using 0.5 mM thenoyltrifluoroacetone. The activity of complex II was calculated based on the linear decrease in 2,6‐dichloroindophenol absorbance at 600 nm over a 10‐min duration.

Complex III activity was measured in an assay mixture containing 25 mM of potassium phosphate (pH 7.6), 50 μM of decyl‐ubiquinol, 2 mM of EDTA, 4 mM of sodium azide, 0.05% of Tween‐20 and 50 μM of cytochrome c. Inhibition of complex III was accomplished using 10 μM antimycin A. The calculation of complex III activity involved measuring the linear increase in cytochrome c absorbance at 550 nm over a 10‐min period.

Freshly prepared decyl‐ubiquinol, obtained by dissolving decyl‐ubiquinone in acidified ethanol at pH 4 and subsequently reducing it with sodium borohydride, was used in the assay for complex IV. Complex IV activity was assessed in an assay mixture comprising 30 mM of potassium phosphate (pH 7.4) and 50 μM of freshly reduced cytochrome c. Inhibition of complex IV was achieved with 4 mM of sodium azide. The activity of complex IV was calculated based on the decrease in cytochrome c absorbance at 550 nm over a 10‐min duration.

### 2.6 ATP Level Analysis

ATP was assayed using a modified version of the enzymatic method described by Piechowiak et al. [22]. Metabolites were extracted from fresh cell lysates by precipitating proteins with 0.6 M of HClO₄ for 1 min in an ice bath. The mixture was then centrifuged at 7500 g for 15 min at 4°C. The resulting deproteinised supernatant was neutralised to a pH of 6.5–6.8 using 2 N KOH/0.5 M triethanolamine, incubated on ice for 30 min and centrifuged again for 15 min to remove any insoluble KOH. This neutralised, deproteinised sample was subsequently used for ATP quantification. For the reaction mixture, 500 μL of 0.1 M triethanolamine buffer containing 0.01 M of MgCl₂, 20 μL of 10 mg/mL NADP (Sigma‐Aldrich), 20 μL of 10 mM glucose, 100 μL of cell lysate and 5 μL of glucose‐6‐phosphate dehydrogenase from *Leuconostoc mesenteroides* (500 U/mg, Sigma‐Aldrich) were added. After a 15‐min incubation in darkness, 5 μL of hexokinase from *Saccharomyces cerevisiae* (350 U/mg, Sigma‐Aldrich) was added to the reaction mixture. Absorbance was measured at 340 nm following an additional 10‐min incubation. An ATP standard (1 mM) was used for calibration, and ATP levels were expressed as micrograms of ATP per microgram of protein in the cell lysate.

### 2.7 Mitochondria Staining

The ΔѰm was analysed using the ΔѰ‐specific stain TMRE (a tetramethyl‐rhodamine ester). The dye selectively labels mitochondria depending on ΔѰm. HT22 cells in all experimental conditions were incubated with 50 nM of TMRE for 30 min at 37°C in 5% CO_2_. After the incubation, the images were captured using a confocal microscopy (Leica Microsystem, Germany).

### 2.8 Isolation and Quantification of DNA From Cytosolic, Mitochondrial and Nuclear Fractions

For each condition, a pellet of ∼4 × 10^6^ HT22 cells was resuspended in 1 mL of DPBS by pipetting and then divided into two 1.5‐mL tubes labelled A and B. Tube A was used for a whole‐cell extract DNA (WCE) to serve as a normal control for the mtDNA measurement from subcellular fractions (tube B). The method for fractionating cells into cytosolic, mitochondrial and nuclear compartments and detecting mtDNA by real‐time qPCR was described by Bryant et al. [23]. Briefly, this protocol utilises three different lysis buffers containing three different detergents (SDS, NP‐40 and Digitonin) and differential centrifugation to gently, yet cleanly, extract DNA from cytosolic, mitochondrial and nuclear pools, minimising cross‐purifications. The DNA is then purified with a phenol/chloroform extraction followed by ethanol precipitation. The concentration and purity of DNA samples were analysed by determining the 260/230 and 260/280 nm absorbance ratios using a NanoVue Plus Spectrophotometer (GE Healthcare, UK). Each DNA fraction was analysed by a quantitative PCR (qPCR) in a 7500 Real‐Time PCR system (Applied Biosystems, Thermo Fisher Scientific Inc., Carlsbad, CA, USA), testing 4.5 ng of DNA in duplicate and in two different runs. PCR reactions were performed in a final volume of 25 μL containing 2× Hot‐Rescue Real‐Time PCR Kit Sybr Green (Diatheva srl, Cartoceto (PU), Italy) and 250 nM of each specific primer to quantify nuclear (TERT gene) and mitochondrial (D‐Loop, control region, non‐coding mtDNA, MT‐CYTB and MT‐RNR2 genes) DNA (Table S1). Cycling parameters were as follows: one cycle of 10 min at 95°C to activate the Hot‐Rescue DNA polymerase followed by 40 cycles in two steps, consisting of 15 s at 95°C and 30 s at 60°C. The fluorescence intensity of the products was measured at the end of each cycle, and post‐PCR melt curve analysis was performed to confirm the specificity of the target. In qPCR, the cycle threshold (Ct) value represents the cycle number at which a sample's reaction crosses a fluorescence threshold, indicating the detection of the target nucleic acid. Lower Ct values indicate higher target sequence amounts, while higher Ct values suggest lower amounts or issues. A PCR negative control (NTC) was tested in each run, always giving no amplification signal for all genes, confirming the accuracy of the PCR experimental procedure. MtDNA abundance relative to nuclear DNA was calculated using the $2^{-{\mathrm{\Delta \Delta C}}_{t}}$ method.

### 2.9 RNA Extraction and RT‐PCR Analysis

RNA extraction from pellets of HT22 cells was performed with the Total RNA Purification Kit (Norgen Biotek Corporation, Thorold, Ontario, Canada) according to the manual instructions. Genomic DNA contamination was removed by an on‐column DNase I digestion during the preparation. For each sample, 500 ng of total RNA (quantified by NanoVue Plus Spectrophotometer) was reverse‐transcribed using the PrimeScript RT Master Mix (Perfect Real Time) (Takara Bio Inc., Shiga, Japan), according to the manufacturer's instructions. The IFNß gene expression was evaluated by Sybr Green real‐time qPCR. The PCR reactions were carried out in duplicate in a final volume of 25 μL containing 2× Hot‐Rescue Real‐Time PCR Kit Sybr Green (Diatheva srl, Cartoceto (PU), Italy) and 200 nM of each primer listed in Table S1. The amplification conditions were as follows: 95°C for 10 min, 40 cycles at 95°C for 15 s, 60°C for 30 s and 72°C for 30 s, followed by a melting curve analysis at the end of each run to exclude the presence of non‐specific products or primer dimers. As a reference gene, GAPDH (glyceraldehyde‐3‐phosphate dehydrogenase) was selected among three candidates (B2M, GAPDH, PPIA). All qPCR reactions were performed in a 7500 Real‐Time PCR system (Applied Biosystems, Thermo Fisher Scientific Inc., Carlsbad, CA, USA). A non‐template control was included in the PCR run as a negative control. The relative expression level of IFNß gene was calculated using the $2^{-{\mathrm{\Delta \Delta C}}_{t}}$ method with Pffaff efficiency correction (95% for GAPDH and 101% for IFNß) [24].

### 2.10 Protein Extraction and Immunoblotting

Subcellular fractions were prepared from HT22 cells according to Bryant et al. [22]. Supernatants were assayed for protein concentration using the Bradford reagent (Sigma‐Aldrich). Protein extracts (5–15 μg) were separated by SDS‐PAGE and transferred to PVDF membranes (Thermo Scientific). A ColorBurst electrophoresis marker (3 μL/gel, Sigma‐Aldrich) was used for qualitative molecular mass determinations and for visual confirmation of blot transfer efficiency. The blots were then blocked with non‐fat dry milk in TBS‐T (10 mM Tris, 150 mM NaCl, pH 7.6, plus 0.1% Tween‐20) and probed overnight at 4°C with the following primary antibodies: anti‐AGC (1:5000, polyclonal; Abcam, ab234975), anti‐ATP synthase subunit α (1:1000, monoclonal; Abcam, ab14748), anti‐ATP synthase subunit β (1:2500, polyclonal; Sigma, HPA001520), anti‐c‐GAS (1:1000, polyclonal; Cell Signalling Technology, #31659), anti‐STING (1:1000, polyclonal; Novus, NBP2‐24683) and anti‐HMGB1 (1:500, polyclonal; Abcam, ab18256). Membranes were washed in TBS‐T and incubated for 60 min with the appropriate secondary antibody diluted to 1:4000 (Santa Cruz Biotechnology), followed by washing in TBS‐T. Proteins were visualised by ECL according to the manufacturer's instructions (RPN2209, Sigma‐Aldrich). A monoclonal antibody against β‐actin (1:4000, Santa Cruz Biotechnology, sc‐8432), a monoclonal antibody against porin (1:1000, Santa Cruz Biotechnology, sc‐390,996) or a polyclonal antibody against Lamin A/C (1:1000, 1:1000, polyclonal; Cell Signalling Technology, #2032) were used as a loading control and for data normalisation. Densitometric analyses were performed using the NIH‐Image J 1.45 software (https://imagej.nih.gov/ij/; National Institutes of Health, Bethesda, MD, USA). Data were expressed as a percentage of control.

### 2.11 Transmission Electron Microscopy (TEM)

HT22 cells were seeded in 75‐cm^2^ flasks at a density of 2 × 10^6^ cells/well and allowed to adhere for 24 h. After treatments, cells were washed, fixed and embedded, as previously described. Ultrastructural analysis was performed with a transmission electron microscope (TEM) at 80 KV (Philips CM10) and imaged with an SIS MegaView II camera (Soft Imaging System) [25]. The analysis of apoptotic mitochondria was performed measuring 20 images for each experimental condition.

### 2.12 ELISA Immunoassays

Supernatants of confluent monolayers of HT22‐treated cells were centrifuged at 2000 g for 5 min, collected and stored at −80°C until assayed. Soluble FGF‐21 and IL‐6 levels from supernatants were assessed by specific immunoassays (DY3057, mouse FGF‐21 DuoSet ELISA and DY406, mouse IL‐6 DuoSet ELISA, respectively) according to the manufacturer's instructions (R&D System, Abingdon, UK).

### 2.13 Statistical Analyses

Quantitative data are expressed as mean ± SD based on at least three independent experiments. Differences between groups were analysed using one‐way analysis of variance (one‐way ANOVA), followed by the Newman–Keuls multiple comparison and Tukey post hoc test. A *p*‐value < 0.05 was considered significant. All statistical analyses were performed using GraphPad Prism 5.0 (GraphPad software).

## 3 Results

### 3.1 Melatonin Reduces Oxidative Cell Damage and Preserves the Mitochondrial Respiratory Chain Activity Affected by OGD/R in HT22 Cells

**FIGURE 1:**
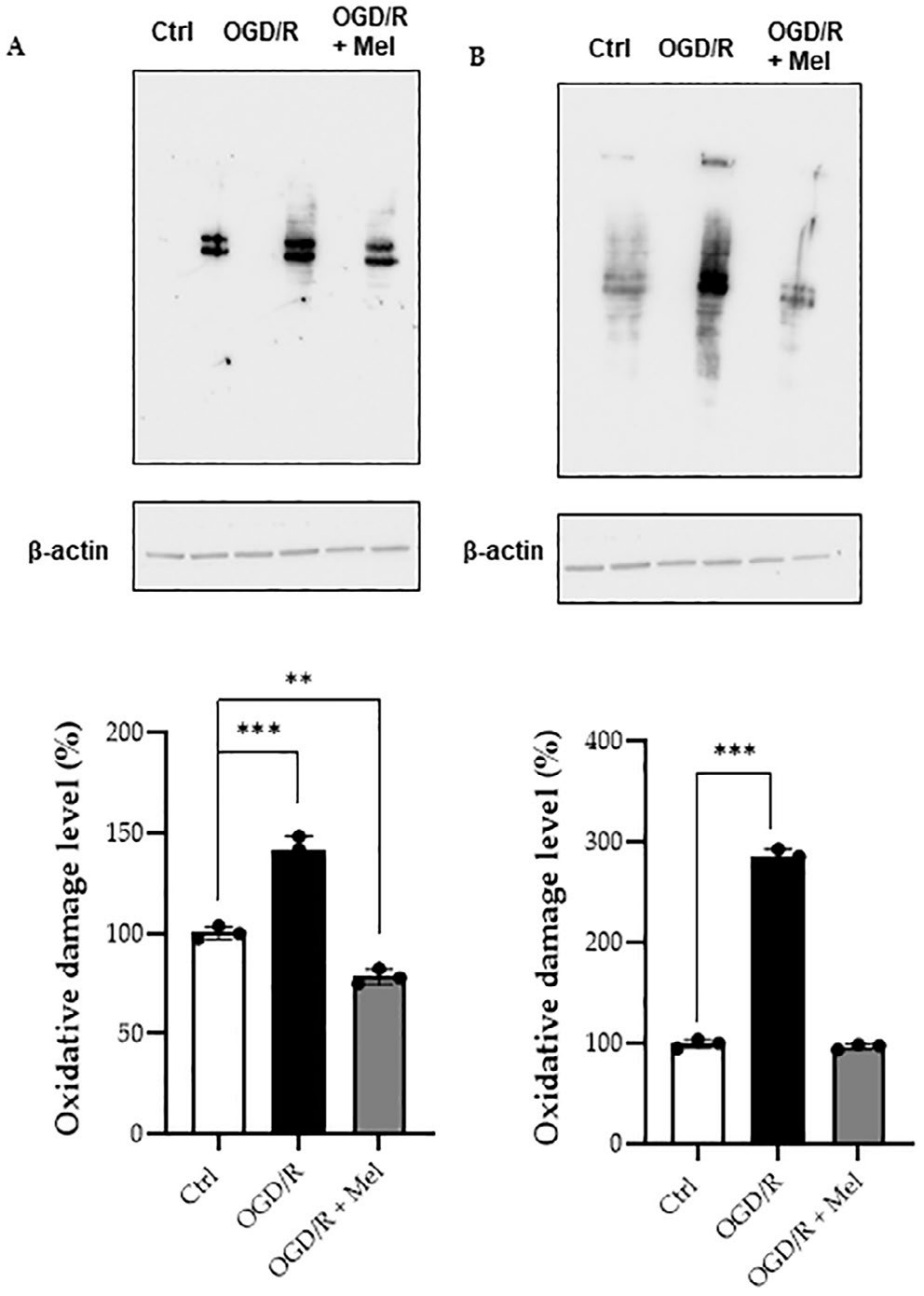
Oxidative damage in HT22 cells after OGD/R and melatonin treatment. (A) Representative Western blots and quantitative evaluation of oxidised proteins (carbonyl group) in whole lysate of untreated HT22 cells (Ctrl), 8‐h OGD‐exposed cells (OGD/R) and 8‐h OGD‐exposed cells in the presence of 50 μM of melatonin (OGD/R + Mel). Analysis was performed after 30 min or 2 h (B) reoxygenation. Data normalised to the loading control ß‐actin are expressed as mean ± SD from at least three independent experiments. **p ≤ 0.01, ***p ≤ 0.001 vs. Ctrl; one‐way ANOVA.

**FIGURE 2:**
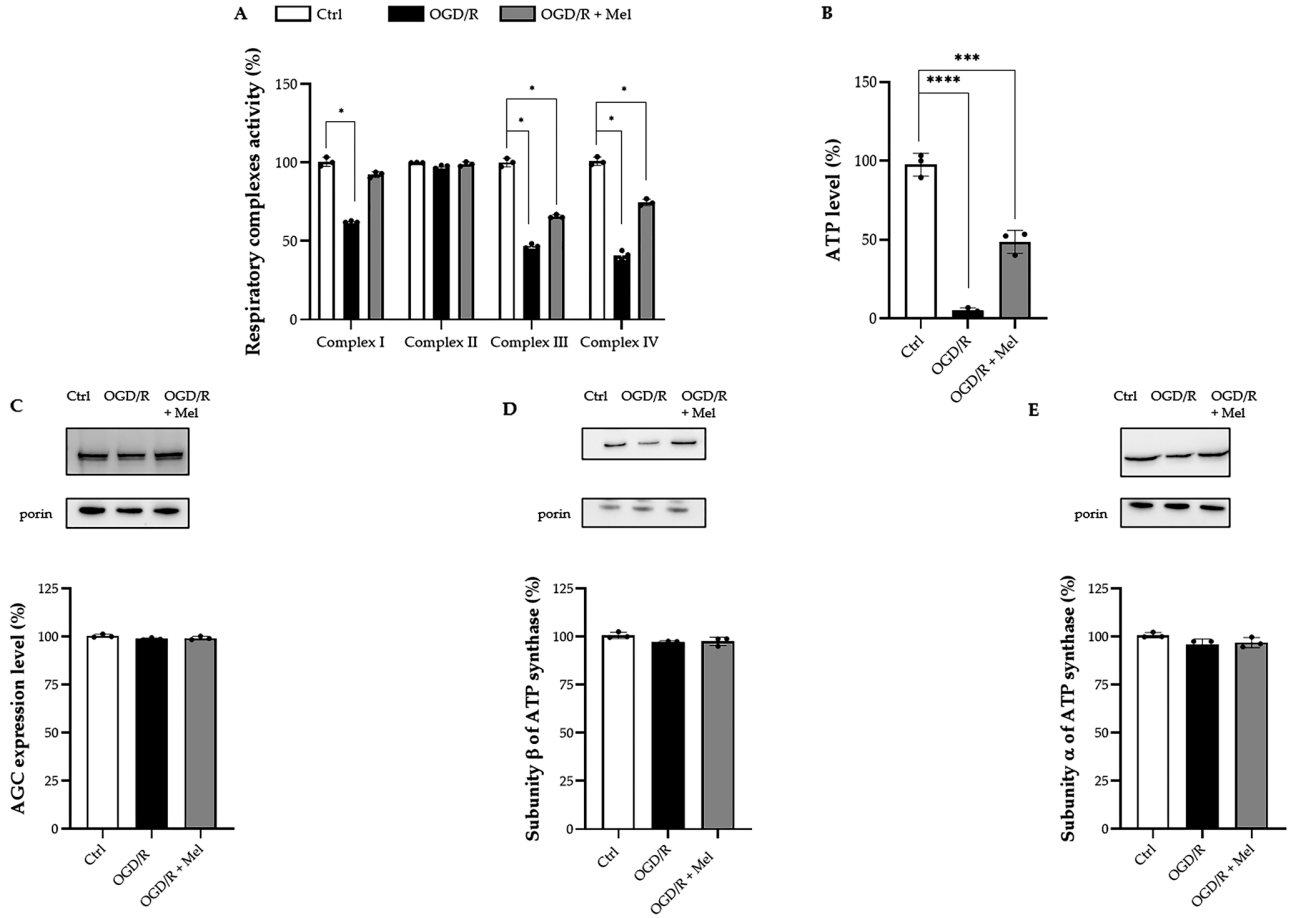
Activity of mitochondrial respiratory chain complexes and ATP level in HT22 cells after OGD/R and melatonin treatment. (A) Enzymatic activity of mitochondrial respiratory chain complexes I, II, III and IV spectrophotometrically measured in untreated HT22 cells (Ctrl), 8‐h OGD‐exposed cells (OGD/R) and 8‐h OGD‐exposed cells in the presence of 50 μM melatonin (OGD/R + Mel). Analysis was performed after 30 min of reoxygenation (R). Data are means ± SD from five independent experiments. Respiratory activities measured for Ctrl were set at 100%. *p ≤ 0.05 vs Ctrl; one‐way ANOVA. (B) ATP level spectrophotometrically measured in untreated HT22 cells (Ctrl), 8‐h OGD‐exposed cells (OGD/R) and 8‐h OGD‐exposed cells in the presence of 50 μM melatonin (OGD/R + Mel). Analysis was performed after 30 min of reoxygenation (R). Data are means ± SD from three independent experiments. ATP levels measured for Ctrl (0,162831 μg of ATP per μg of protein in the cell lysate) were set at 100%. ****p ≤ 0.0001, ***p ≤ 0.001 vs. Ctrl; one‐way ANOVA. (C) Representative Western blot and quantitative evaluation of aspartate/glutamate carrier (AGC), subunit β (D) and subunit α (E) of ATP synthase expression examined in isolated mitochondria from Ctrl, OGD/R and OGD/R + Mel cells performed after 30 min of reoxygenation. Data normalised to the loading control porin are expressed as % of Ctrl and are the mean ± SD (*N* = 3 independent experiments performed in triplicate). No statistical difference was detected among experimental groups through the ANOVA test.

Carbonyl formation by the Oxyblot analysis was used to assess the effects of OGD/R and melatonin on protein oxidation. As shown in Figure 1, increased levels of carbonylated proteins were observed in OGD/R cells 30 min after reoxygenation compared to the control condition (Figure 1A). Accumulation of oxidatively modified proteins was also found 2 h after injury (Figure 1B). Melatonin significantly reduced protein carbonyl formation at both the time points analysed (Figure 1). To assess if the increased oxidative stress affected the mitochondrial function, we measured the activity of the mitochondrial respiratory chain complexes I, II, III and IV. OGD/R significantly decreased the complex I, III and IV enzymatic activities by 40%, 55% and 60%, respectively. In contrast, no effect was observed on complex II activity (Figure 2A). In the presence of melatonin, complex I activity was almost completely recovered, whereas a lower effect, although not significant, was found for the recovery of the activities of complexes III and IV. No effect of melatonin was observed on complex II activity (Figure 2A). OGD/R significantly reduced the ATP production by approximately 95% compared to control. In the presence of melatonin, ATP production was partially restored, reaching about 50% of the control (Figure 2B). Since the subunits of complexes I, III and IV are mostly encoded by mtDNA [26] and those of complex II are encoded by nuclear DNA [27], we evaluated the expression of encoded nuclear DNA proteins involved in the transport of metabolites and in the oxidative phosphorylation process, i.e., aspartate/glutamate carrier (AGC) and α and β subunits of ATP synthase [28, 29]. Immunoblot analysis revealed that neither OGD/R nor melatonin affected the expression of these proteins (Figure 2C–E), suggesting that OGD/R and melatonin effects primarily involved mtDNA.

### 3.2 Melatonin Preserves the Mitochondrial Structure Affected by OGD/R

**FIGURE 3:**
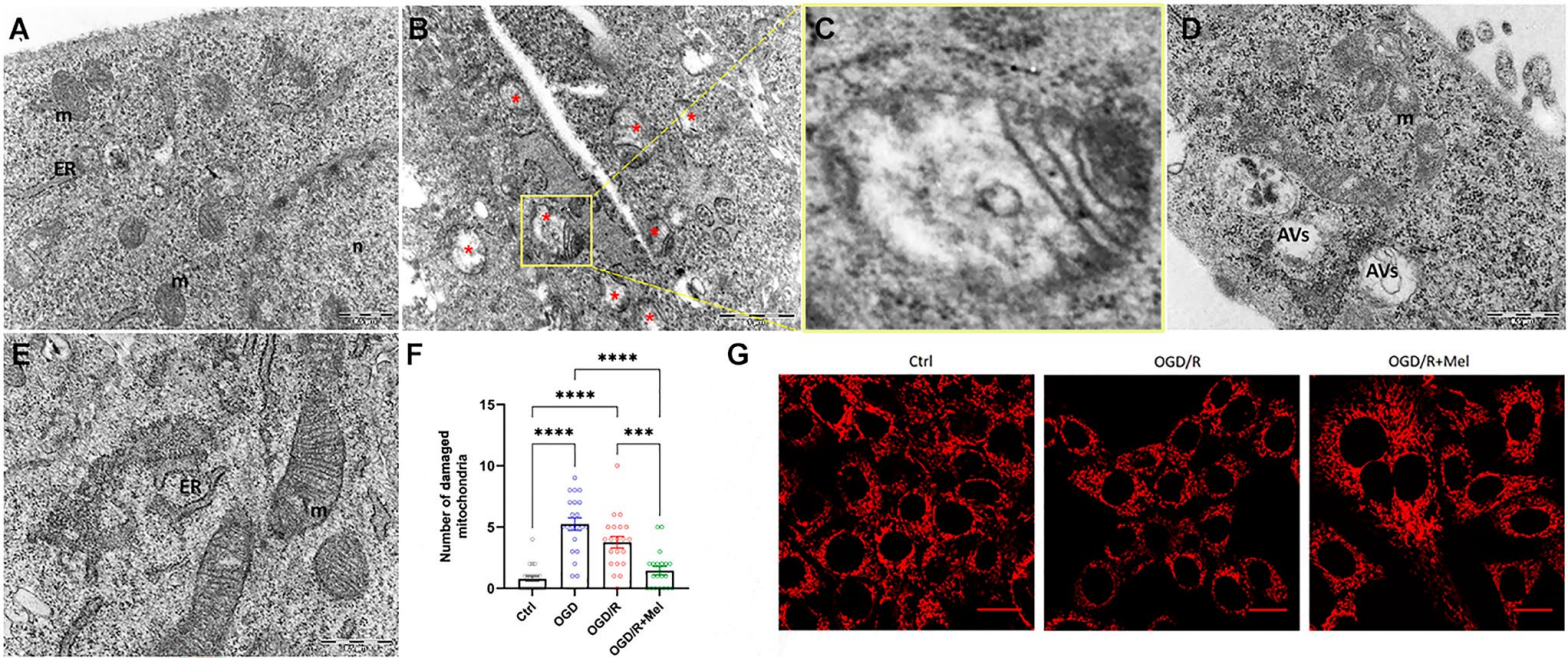
Mitochondrial analysis in HT22 cells after OGD/R and melatonin treatment. Electron microscopy images (A–E) and confocal microscopy images (G, H) of untreated HT22 cells (Ctrl), 8‐h OGD‐exposed cells (OGD) or followed by 2 h of reoxygenation (OGD/R) and 8‐h OGD‐exposed cells followed by 2 h of reoxygenation in the presence of 50 μM of melatonin (OGD/R + Mel). Images show typical mitochondria tubular cristae with normal shapes and sizes in Ctrl cells (A) and numerous damaged mitochondria (red asterisks) with typical electron‐dense inclusions in OGD cells (B), as shown in the enlarged inset (C). Damaged mitochondria, with swollen, disrupted cristae in a low‐density matrix, are shown in OGD/R condition (D); conversely, healthy mitochondria with a dense matrix and clear cristae structure are shown in OGD/R + Mel cells (E). (F) Damaged mitochondria were quantified by analysing 20 images for each experimental condition. Data are expressed as mean ± SEM ***p < 0.01; ****p < 0.0001; one‐way ANOVA. Panels (G) show confocal microscopy images of mitochondrial membrane potential (TMRE) labelling in Ctrl, OGD/R and OGD/R + Mel cells. Scale bar, 25 μm. AVs, autophagic vacuoles; ER, endoplasmic reticulum; m, mitochondria; n, nucleus.

TEM was performed to evaluate the condition of the mitochondria. Control cells showed normal‐shaped and normal‐sized mitochondria with typical tubular cristae (Figure 3A). In contrast, in OGD cells, we observed several structurally altered mitochondria with electron‐dense inclusions both before (OGD; Figure 3B,C) and after reoxygenation (OGD/R; Figure 3D). OGD/R cells also showed autophagic vacuoles with autophagosome‐like features, indicating a concomitant activation of the autophagy process (Figure 3D) as previously observed [30]. Mitochondria in OGD/R + Mel cells, in contrast, appeared better preserved with a dense matrix and clear cristae structure (Figure 3E). Furthermore, the quantitative TEM analysis of healthy mitochondria (i.e., with dense matrix and clear cristae structure) and damaged mitochondria (with swollen and disrupted cristae in a low‐density matrix) revealed that the number of damaged mitochondria was significantly decreased in melatonin‐treated cells compared to both OGD and OGD/R HT22 cells (Figure 3F). In keeping with TEM analysis, OGD/R + Mel cells showed more TMRE‐positive mitochondria than OGD/R cells, whose mitochondria are characterised by structural changes from a tubular to a globular shape (Figure 3G).

### 3.3 Melatonin Reduces the mtDNA Cytosolic Efflux and Stimulates FGF‐21 Release by HT22 Cells During OGD/R

**FIGURE 4:**
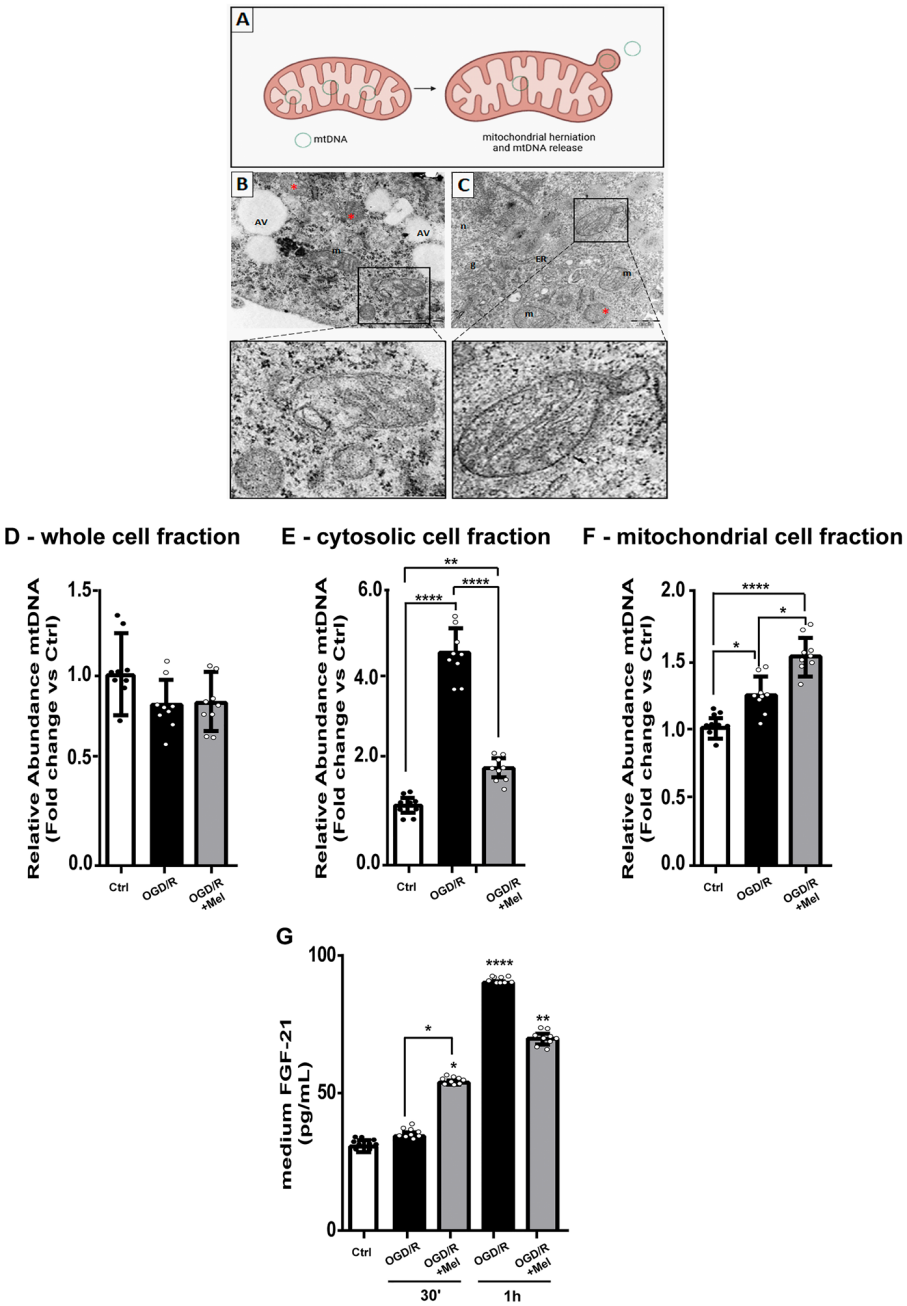
Mitochondrial DNA (mtDNA) analysis in HT22 cells after OGD/R and melatonin treatment. (A) Schematic representation of mitochondrial herniations, created by BioRender.com. (B) Electron microscopy imaging of 8‐h OGD‐exposed cells followed by 2 h of reoxygenation (OGD/R) and 8‐h OGD‐exposed cells in the presence of 50 μM of melatonin (OGD/R + Mel, C) show the presence of apoptotic mitochondria (red asterisks) and mitochondrial herniation. The area insets show a higher magnification of mitochondria during herniation and release of bound vesicles in OGD/R and OGD/R + Mel cells. AVs, autophagic vacuoles; ER, endoplasmic reticulum; g; Golgi apparatus; m, mitochondria; n, nucleus. (D) mtDNA analysis in whole‐cell, cytosolic (E), and mitochondrial (F) extracts of untreated HT22 cells (Ctrl), OGD/R and OGD/R + Mel cells performed after 30 min of reoxygenation. Data are mean ± SD from three mitochondrial genes quantified by qPCR, two runs for each gene.*p < 0.05, **p < 0.01, ****p < 0.0001; one‐way ANOVA. (G) FGF‐21 levels were measured in the medium of Ctrl, OGD/R and OGD/R + Mel cells after 30 min and 1 h of reoxygenation. Data are mean ± SD (*N* = 3 independent experiments performed in triplicate). *p < 0.05, **p < 0.01, ****p < 0.0001 vs. Ctrl, *p < 0.05, bars; Tukey's multiple comparison test.

TEM experiments showed herniations of the outer mitochondrial membrane into the cytosol of OGD/R and OGD/R + Mel cells (Figure 4B,C). Mitochondrial herniations indicate outer mitochondrial membrane permeabilisation and macropore formation, with the inner mitochondrial membrane ballooning out into the cytoplasm carrying mtDNA and proteins (Figure 4A) [31]. Thus, we analysed the mtDNA content and location after ODG/R and melatonin treatment to assess its release. Preliminarily, we tested nuclear DNA contamination in mitochondrial and cytosolic extracts using the nuclear TERT gene amplification. Low or no TERT amplification (Ct values > 30) was found, indicating irrelevant nuclear contamination in our experimental conditions. The total amount of mtDNA in whole‐cell extracts did not differ among the experimental groups (Figure 4D). After 30 min of reperfusion, mtDNA in the cytosol of OGD/R cells was present in significant amounts (Figure 4E). The amount of cytosolic mtDNA was significantly lower in OGD/R cells treated with melatonin (Figure 4E; *p* ≤ 0.0001 vs. OGD/R). The parallel analysis of the mitochondrial fractions showed that mtDNA was slightly increased in OGD/R cells compared to the control condition (Figure 4F; *p* ≤ 0.05). A much higher amount of mtDNA was observed in the mitochondria of OGD/R cells treated with melatonin (Figure 4F; *p* ≤ 0.0001 and *p* ≤ 0.05, compared to the control and OGD/R condition, respectively). Besides, we observed the release of the key metabolic mediator FGF‐21 in the medium in the presence of melatonin that was significantly reduced after 1 h of reoxygenation compared to OGD/R condition (Figure 4G).

### 3.4 Melatonin Reduces the Activation of the mtDNA‐Induced cGAS/STING Pathway During OGD/R in HT22 Cells

**FIGURE 5:**
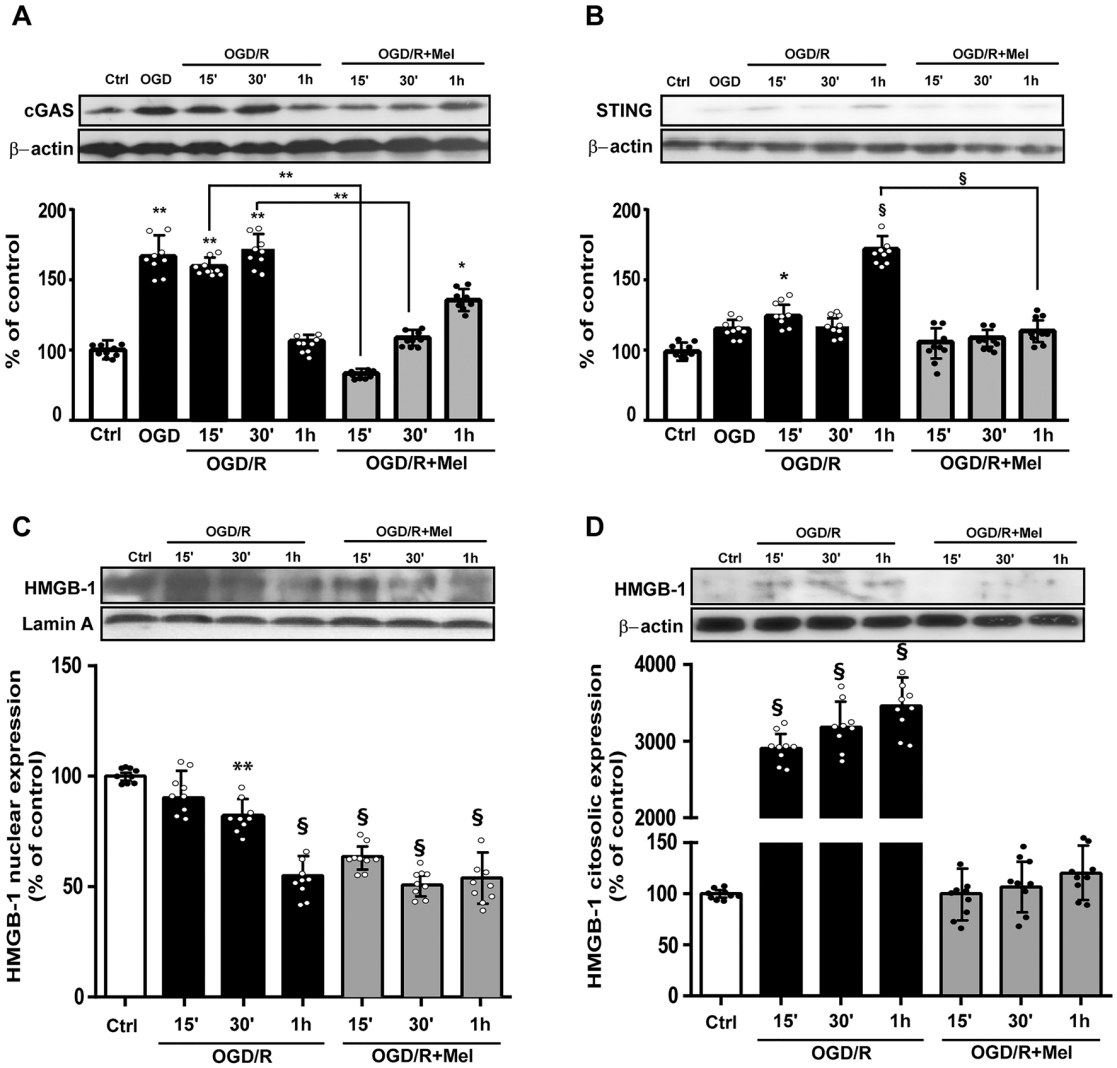
cGAS, STING and HMGB‐1 expressions in HT22 cells after OGD/R and melatonin treatment. (A) Quantitative evaluation and representative Western blots of cGAS, STING (B) and nuclear (C) and cytosolic (D) HMGB‐1 expressions in untreated HT22 cells (Ctrl), 8‐h OGD‐exposed cells (OGD) or followed by 15 min, 30 min or 1 h of reoxygenation (OGD/R) and 8‐h OGD‐exposed cells followed by 15 min, 30 min or 1 h of reoxygenation in the presence of 50 μM melatonin (OGD/R + Mel). Data normalised to the loading control ß‐actin or lamin A are expressed as % of control and are the mean ± SD (*N* = 3 independent experiments performed in triplicate); *p < 0.05, **p < 0.01, ^§^ *p* < 0.001 vs Ctrl; **p < 0.01, ^§^ *p* < 0.001, bars.

Mitochondrial stress and mtDNA release into the cytosol are closely associated with the activation of the cGAS–STING pathway and neuroinflammatory responses [9]. cGAS is a key cytosolic mtDNA sensor, and therefore, we analysed its expression. cGAS expression significantly increased after OGD (Figure 5A). The increased expression of the protein was found up to 1 h after reoxygenation (Figure 5A). Melatonin significantly reduced cGAS protein expression at all the time points evaluated (Figure 5A). STING expression was barely detectable in control conditions, slightly increased 15 and 30 min after OGD and showed the highest increase after 1 h of reperfusion (Figure 5B). Melatonin completely blocked its increase (Figure 5B). Melatonin also reduced the release of HMGB‐1, a protein passively released during cellular stress. OGD/R significantly and time‐dependently reduced the HMGB‐1 expression in the nuclear fraction (Figure 5C). In parallel with this change, its expression increased in the cytosol after OGD/R (Figure 5D). Melatonin significantly reduced the HMGB‐1 expression in both the nuclear and cytosolic fractions (Figure 5C,D).

### 3.5 Melatonin Reduces Interferon Beta (IFNβ) mRNA Expression and IL‐6 Release During OGD/R in HT22 Cells

**FIGURE 6:**
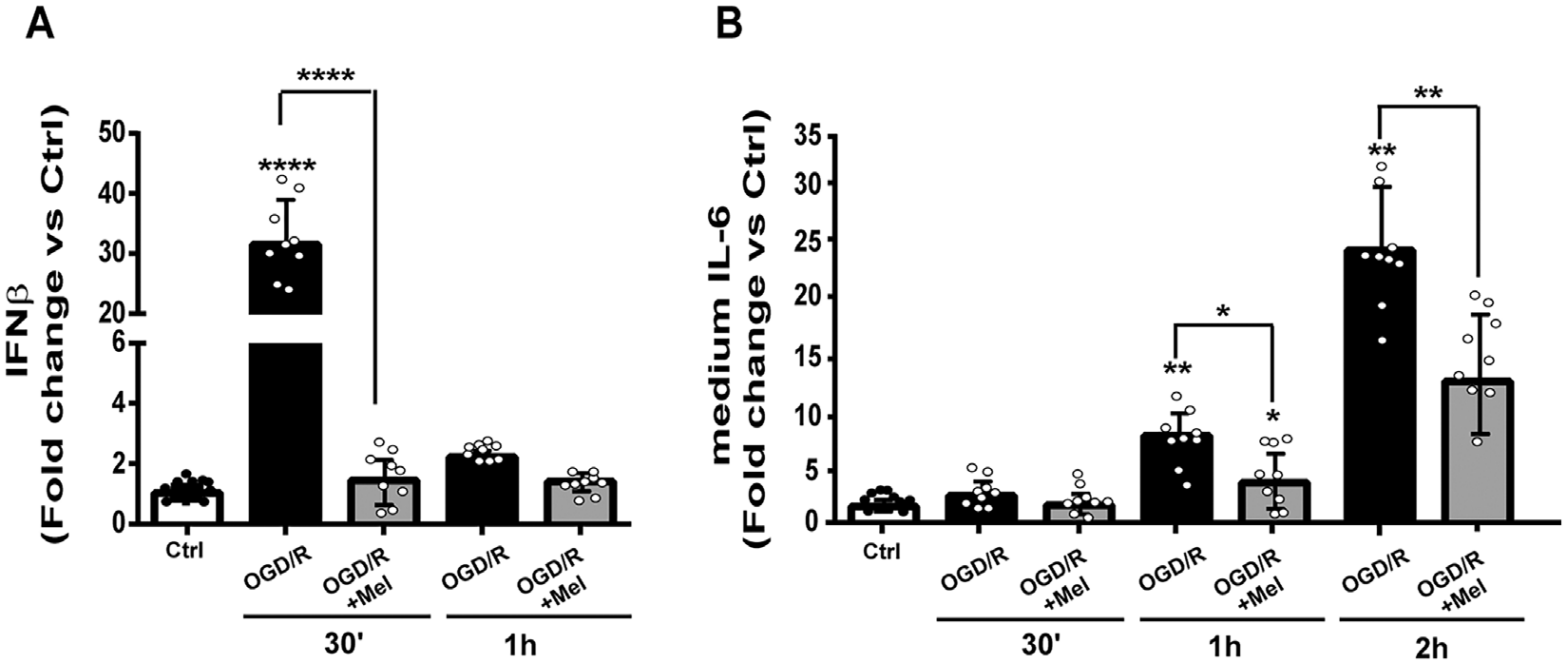
IFNβ mRNA expression and IL‐6 release from HT22 cells during OGD/R and melatonin treatment. (A) Interferon beta (IFNβ) mRNA expression in untreated HT22 cells (Ctrl), 8‐h OGD‐exposed cells (OGD/R) and 8‐h OGD‐exposed cells in the presence of 50 μM of melatonin (OGD/R + Mel). Analysis was performed after 30 min and 1 h of reoxygenation. The data were normalised to the housekeeping gene encoding GAPDH and are expressed as mean ± SD. *p < 0.05, **p < 0.01, ****p < 0.001; one‐way ANOVA. Genes were considered to be up‐regulated or down‐regulated if changes in their expression levels were ≥ 2‐fold or ≤ 2‐fold, respectively. (B) Interleukin‐6 (IL‐6) levels were measured in the medium of Ctrl, OGD/R and OGD/R + Mel cells after 30 min, 1 h and 2 h of reoxygenation. Data are mean ± SD (*N* = 3 independent experiments performed in triplicate). *p < 0.05, **p < 0.01 vs. Ctrl, *p < 0.05, **p < 0.01, bars; Tukey's multiple comparison test.

The modulation of the mtDNA‐cGAS/STING pathway observed after OGD/R and melatonin treatment was further analysed by measuring the IFNβ mRNA expression and the IL‐6 release into the cell culture medium. OGD/R significantly increased the IFNβ mRNA levels, inducing more than a 30‐fold increase compared to Ctrl cells (Figure 6A, *p* < 0.001); after melatonin treatment, the IFNβ gene expression returned to control levels (Figure 6A). The IL‐6 release into medium showed a similar pattern of IFNβ. As shown in Figure 6B, the OGD/R insult significantly increased the IL‐6 levels compared to Ctrl cells; melatonin significantly reduced these OGD/R effects (Figure 6B).

## 4 Discussion

Mitochondria are complex organelles playing a central role in energy metabolism and stress responses and are hubs for biosynthetic processes. Consistent with their multifunctional role, mitochondrial dysfunctions are key events in many pathological conditions, including neurodegenerative diseases [32]. We previously reported that OGD/R significantly affected the mitochondrial network in HT22 cells. Melatonin preserved the mitochondrial network and improved cell survival [18]. Herein, we extended those observations by assessing mito‐inflammation after OGD/R and its modulation by melatonin. We report that melatonin preserves the mitochondrial structure and function affected by OGD/R. In our experimental conditions, melatonin reduced oxidative damage, preserved the mitochondrial respiratory chain activity and reduced mtDNA cytosolic release and the cGAS–STING pathway‐related sterile inflammatory response. Analysis of the mitochondrial respiratory chain activity after OGD/R revealed decreased enzymatic activities for complexes I, III and IV during reoxygenation. No effects were observed on complex II activity. Since complexes I, III and IV are encoded by mtDNA, whereas complex II is encoded by nuclear DNA, our results suggest that the mitochondrial dysfunction observed after OGD/R may primarily result from ROS interactions with mtDNA. In line with this hypothesis are the reduction in protein carbonylation and the recovery of the enzymatic activity of mtDNA‐encoded OXPHOS complexes following melatonin administration. The assessment of ATP levels, in addition, revealed that OGD/R severely compromised ATP production, indicating a significant impairment of mitochondrial function, as both oxygen and glucose are essential for efficient ATP production by OXPHOS. In our experiments, the α and β subunits of the FoF1‐ATP synthase complex encoded by nuclear DNA did not show altered expression despite the reduction in ATP levels. This suggests that the mitochondrial dysfunction caused by impaired activity of mtDNA‐encoded complexes could be the leading cause of reduced ATP production. Melatonin partially restores ATP production, correlating with mitochondrial function and complex activity recovery. It should also be noted that neither OGD/R nor melatonin affected the expression of other mitochondrial components encoded by nuclear DNA and involved in metabolite transport or OXPHOS processes [28, 29], such as the mitochondrial aspartate/glutamate carrier (Figure 2C).

Our results also show that OGD/R significantly induced the mtDNA release from mitochondria into the cytosol in the early reoxygenation phase. Previous studies have shown the critical importance of mtDNA in maintaining mitochondrial function under stress conditions [33]. Damage to mtDNA and its release into the cytosol during reperfusion were observed in the heart, liver and kidney, in various ischaemic damage models, suggesting that mtDNA damage is an important component of the injury development [34]. In our experiments, melatonin reduces mtDNA release into the cytosol, suggesting that it can preserve mtDNA and maintain mitochondrial integrity. Melatonin possesses scavenging effects on ROS/RNS production, inhibits the mPTP and activates uncoupling proteins, which helps maintain the optimal mitochondrial membrane potential [34]. Furthermore, melatonin also works as an indirect antioxidant by preventing cytosolic calcium overload [35]. All these effects are crucial for reducing oxidative stress and consequently mtDNA damage and its release in the cytosol. The reduction of the protein carbonyl content showed herein and our previous findings showing that melatonin significantly reduced mitochondrial ROS production [18] are in line with our hypothesis. Nevertheless, further research is needed to determine if the reduction of mtDNA release into the cytosol is a direct effect of the reduction of ROS formation or is a consequent to melatonin‐induced modifications on signalling pathways inside the mitochondria, such as, for example, its effect on SIRT3 [18].

Cytosolic mtDNA acts as a DAMP, stimulates the cGAS–STING pathway and activates the inflammatory response by increasing the release of inflammatory cytokines. By reducing the upstream mtDNA release, melatonin lessens the cGAS/STING pathway activation and decreases the sterile inflammation response. mtDNA release and cGAS–STING activation have also been observed in other stress conditions, including in the aging brain of Alzheimer's disease mice [36, 37], in amyotrophic lateral sclerosis condition followed by inflammatory cytokine secretion [38] and in ischaemic/reperfusion injury [39]. This was concomitant to the cytosolic release of the protein HMGB‐1 from the nucleus, another key event in the pathogenesis of sterile inflammation [40]. Our results are consistent with those reported by Jauhari et al. [36], who observed that melatonin reduced the release of mtDNA in the striatum of HD mice, inhibiting the activation of the cGAS–STING pathway and the expression of pro‐inflammatory cytokines, leading to neuroprotection.

Besides preserving the mitochondrial respiratory chain activity, melatonin also modulated the release of FGF‐21. OGD/R induced FGF‐21 release after 1 h of reoxygenation. Melatonin anticipated the release of the mitokine since we found a significant increase after 30 min of reoxygenation when we did not observe the release in OGD/R condition and reduced its release at 1 h compared to the OGD/R condition. We do not have a clear explanation for this dual effect of melatonin. We hypothesise that the reduced release observed after 1 h of reoxygenation may result from the melatonin's protective effects and the reduced activation of the inflammatory cGAS–STING pathway. The early release of FGF‐21 may contribute to acclimating distant cells to the stressful environment, stimulating homeostatic mechanisms, and can be part of an integrated response to the mitochondrial dysfunction that spreads to tissues distant from the damage site. The release of FGF‐21 was previously observed in mitochondrial disorders that impaired OXPHOS and diminished ATP production [41], and administration of the recombinant human FGF‐21 alleviated neuroinflammation and ischaemic brain damage in the middle cerebral artery occlusion (MCAO) rat model [42] and after neonatal brain ischaemia [43]. In addition, this mitokine protects myocardial cells against ischaemic injury [44].

In summary, our results show that melatonin reduces sterile inflammation in HT22 cells exposed to OGD/R and promotes the FGF‐21 release from cells, highlighting the key role of mitochondria in the protective effects of melatonin against ischaemic brain injury. How the effects of melatonin are linked to its ROS scavenging actions or other upstream mechanisms requires further investigation.

## Author Contributions

**Silvia Carloni:** conceptualization (equal), data curation (equal), methodology (equal), supervision (equal), writing – original draft (equal). **Maria Gemma Nasoni:** conceptualization (equal), data curation (equal), methodology (equal), supervision (equal). **Anna Casabianca:** data curation (equal), methodology (equal). **Chiara Orlandi:** investigation (equal), methodology (equal). **Loredana Capobianco:** methodology (equal), writing – original draft (equal). **Giorgia Natalia Iaconisi:** investigation (equal), methodology (equal). **Sabrina Burattini:** methodology (equal). **Serena Benedetti:** investigation (equal), methodology (equal). **Russel J. Reiter:** validation (equal), writing – review and editing (equal). **Walter Balduini:** project administration (equal), supervision (equal), validation (equal), writing – original draft (equal), writing – review and editing (equal). **Francesca Luchetti:** conceptualization (equal), project administration (equal), supervision (equal), validation (equal), writing – original draft (equal), writing – review and editing (equal). **Liana Cerioni:** conceptualization (equal).

## Ethics Statement

The authors have nothing to report.

## Consent

The authors have nothing to report.

## Conflicts of Interest

The authors declare no conflicts of interest.

## Supporting information


**Table S1.** Primers used in the study.

## Data Availability

Data available on request from the authors.
