# Supplementary material for: Melatonin Reduces Mito‐Inflammation in Ischaemic Hippocampal HT22 Cells and Modulates the cGAS–STING Cytosolic DNA Sensing Pathway and FGF21 Release
Source: J Cell Mol Med. 2024 Dec 20;28(24):e70285. doi: 10.1111/jcmm.70285 (PMC11662137; doi:10.1111/jcmm.70285)
Supplement: Supplementary file 1 — Table S1. Primers used in the study. [file JCMM-28-e70285-s001.docx]

**Supplemental Table 1**. Primers used in the study

| Name | Use | Forward sequence 5’-3’ | Reverse sequence 5’-3’ | Reference |
| --- | --- | --- | --- | --- |
| TERT | Nuclear DNA control | CTAGCTCATGTGTCA  AGACCCTCTT | GCCAGCACGTTTC  TCTCGTT | Bryant JD, Current protocols 2022; e372 https://doi.org/10.1002/cpz1.372 |
| MT-DLoop | mtDNA | AATCTACCATCCTCC  GTGAAACC | TCAGTTTAGCTA  CCCCCAAGTTTAA | Bryant JD, Current protocols 2022; e372 https://doi.org/10.1002/cpz1.372 |
| MT-CYTB | mtDNA | GCTTTCCACTTCA  TCTTACCATTTA | TGTTGGGTTGTTTG  ATCCTG | Bryant JD, Current protocols 2022; e372 https://doi.org/10.1002/cpz1.372 |
| MT-RNR2 | mtDNA | CTAGAAACCCCG  AAACCAAA | CCAGCTATCACCAA  GCTCGT | Bryant JD, Current protocols 2022; e372 https://doi.org/10.1002/cpz1.372 |
| GAPDH | Gene expression | TGCCCCCATGTTTGT  GATG | TGTGGTCATGAG  CCCTTCC | FASEB BioAdvances. 2019; 1:296–305 https://doi.org10.1096/fba.2018-00066 |
| IFNß | Gene expression | CTGCGTTCCTGCTGT  GCTTCTCCA | TTCTCCGTCAT  CTCCATAGGGATC | Chamma H et al., STAR Protocols 2022; 3, 101384 https://doi.org/10.1016/j.xpro.2022.101384 |
